# Dabie bandavirus infection induces macrophagic pyroptosis and this process is attenuated by platelets

**DOI:** 10.1371/journal.pntd.0011488

**Published:** 2023-07-24

**Authors:** Sicong Yu, Qinyi Zhang, Lingxuan Su, Ji He, Wen Shi, Hao Yan, Haiyan Mao, Yi Sun, Dongqing Cheng, Xuan Wang, Yanjun Zhang, Lei Fang

**Affiliations:** 1 Department of Critical Care Medicine, Sir Run Run Shaw Hospital, College of Medicine, Zhejiang University, Hangzhou, China; 2 School of Medical Technology and Information Engineering, Zhejiang Chinese Medical University, Hangzhou, China; 3 The First People’s Hospital of Xiaoshan District, Hangzhou, China; 4 Department of Microbiology, Zhejiang Provincial Center for Disease Control and Prevention, Hangzhou, China; 5 Blood Center of Zhejiang Province, Hangzhou, China; 6 Shaoxing Shangyu District Center for Disease Control and Prevention, Shaoxing, China; 7 Key Laboratory of Microbial Technology and Bioinformatics of Zhejiang Province, Hangzhou, China; Beijing Children’s Hospital Capital Medical University, CHINA

## Abstract

Severe fever with thrombocytopenia syndrome (SFTS) is an emerging tick-borne infection with a high mortality rate in humans, which is caused by Dabie bandavirus (DBV), formerly known as SFTS virus. Clinical manifestations of SFTS are characterized by high fever, thrombocytopenia, leukopenia, hemorrhage, gastrointestinal symptoms, myalgia and local lymph node enlargement with up to 30% case fatality rates in human. Macrophage depletion in secondary lymphoid organs have important roles in the pathogenic process of fatal SFTS, but its exact cell death mechanism remains largely unknown. Here, we showed for the first time that DBV infection induced macrophagic pyroptosis, as evidenced by swollen cells, pore-forming structures, accumulation of gasdermin D N-terminal (GSDMD-NT) as well as the release of lactate dehydrogenase (LDH) and IL-1β in human macrophages. In addition to the upregulation of pyronecrosis genes, the expressions of pyroptosis-related proteins (GSDMD, caspase-1 and IL-1β) were also elevated. To be noted, platelets were found to play a protective role in DBV-derived pyroptosis. Transcriptome analysis and *in vitro* studies demonstrated that platelets significantly reduced the gene expressions and protein production of pro-pyroptotic markers and inflammatory cytokines in macrophages, whereas platelets conferred a propagation advantage for DBV. Collectively, this study demonstrates a novel mechanism by which DBV invasion triggers pyroptosis as a host defense to remove replication niches in human macrophages and platelets provide an additional layer to reduce cellular death. These findings may have important implications to the pathogenesis of lethal DBV, and provide new ideas for developing novel therapeutics to combat its infection.

## Author summary

Dabie bandavirus (DBV) is an emerging tick-borne virus with up to 30% case fatality rates in human. Macrophage exhaustion and cytokine storms are essential for the development of fatal SFTS. However, the mechanisms by which DBV induces macrophagic cell death and causes excessive inflammatory response remain poorly explored. Here, we first demonstrate caspase-1-mediated pyroptosis in human macrophages, a formerly unknown mechanism to facilitate hose defense against DBV infection. DBV-induced pyroptotic cell death was evidenced by pore-forming structures, increased release of LDH and expression of pyroptosis-related proteins. More importantly, platelets protected macrophages from pyroptosis, by attenuating the gene expressions of pyroptosis-related markers and inflammatory cytokines in macrophages. Taken together, our study provides unique perspective on macrophagic cell death pathways in SFTS and identifies a novel function of platelets, which may provide potential treatment options for DBV-related diseases.

## Introduction

Severe fever with thrombocytopenia syndrome (SFTS) is an emerging hemorrhagic fever caused by Dabie bandavirus (SFTS virus), a novel banda-virus in the family *Phenuiviridae* and the order *Bunyavirales* [1,2]. This disease is endemic to central and eastern China [3], South Korea [4], Japan [5], and poses a substantial risk to public health [6]. Clinical manifestations of SFTS are characterized by high fever, thrombocytopenia, leukopenia, hemorrhage, gastrointestinal symptoms, myalgia and local lymph node enlargement with up to 30% case fatality rates [7]. Patients with severe SFTS usually develop lymphadenopathy, bleeding tendency, central nervous system symptoms and multiple organ dysfunction, leading to poor prognosis and fatal outcomes [8]. Medical countermeasures against this devastating disease remain limited. Due to its epidemic potential and high clinical mortality, World Health Organization has prioritized SFTS as a key disease that requires rigorous research and more testing [7].

In SFTS, inflammatory cytokine storms and impairment of immune responses have a significant impact on disease severity [9–12]. Immune cells such as macrophages in secondary lymphoid organs are believed to be the primary targets for DBV infection in fatal SFTS [13,14]. Although they play a critical role in viral eradication, macrophages are susceptible to DBV replication [15,16]. Ferret animal model has revealed that impaired macrophagic functions and progressive viral propagation were associated with DBV-induced mortality [17]. However, the exact mechanisms underlying programmed cell death (PCD) of macrophages during DBV infection remain elusive.

Viral-induced PCD mainly includes apoptosis, pyroptosis and necroptosis [18–20]. It has been found that DBV-infected macrophages were almost free of apoptosis [13,21], suggesting macrophagic PCD might be regulated by other pathways in SFTS. Distinct from apoptosis, pyroptosis is a cytolytic programmed cell death that triggers inflammatory responses to resist microbial infections. During pyroptosis, caspases are activated by many inflammasomes such as NLRP3 [22] and the N-terminal domain forms holes in the cell membrane, leading to the rupture of the plasma membrane and the release of interleukins (IL)-1β and IL-18 [23].

Previous studies demonstrated the expression of and secretion of IL-1β and macrophage inflammatory protein 1β in fatal SFTS cases [10,24]. A recent study found that inflammasome NLRP3, a key player in antiviral responses, was also observed in DBV-infected macrophages, which may facilitate IL-1β and IL-18 secretion and induce pyroptosis [16,25]. These features suggest that pyroptosis might participate in the pathophysiology of SFTS. Whether pyroptosis is involved in DBV-triggered cell death of macrophages and leads to disease severity, however, has not been examined.

In this study, we elucidated the mechanisms by which DBV infection induced pyroptosis-mediated cell death of human macrophages. Our *in vitro* data revealed cell swollen, GSDMD-mediated pore-forming structure, cytoplasmic efflux and cytokine secretion in human macrophages upon DBV exposure. Further, the transcription of pyroptotic genes and the translation of pyroptotic proteins were significantly up-regulated in macrophages. It is of great interest that platelets repressed pyroptotic cell death but enhanced DBV replication in human macrophages. These findings provide unique perspectives on host immune response to eradicate DBV as an antiviral defense in SFTS and potentially therapeutic implications for its treatment.

## Material and methods

### Ethics statement

The studies using human blood were reviewed and approved by the Ethics Committee of Zhejiang Provincial Center for Disease Control and Prevention, China. Blood collection was carried out according to the guidelines and regulations. All donors provided their written informed consent to participate in this study.

### Cell culture and virus

THP-1 cell line is derived from the peripheral blood of human monocytic leukemia with reproducible treatment responses, which has been commonly used as a monocyte proxy for research purposes [26]. Human monocytic cell line THP-1 (Meisen, Chinese Tissue Culture Collections, Zhejiang, China) was derived from the peripheral blood of human monocytic leukemia with reproducible treatment responses and cultured in Roswell Park Memorial Institute 1640 medium (RPMI 1640, Gibco, USA) containing 10% heat-inactivated fetal bovine serum (FBS, Gibco, USA) and 1% penicillin-streptomycin solution (PS, Gibco, USA) at 37°C with an atmosphere of 5% CO_2_. THP-1 cells were differentiated into resting (M0) macrophages by the stimulation of phorbol-12-myristate-13-acetate (PMA, 100 ng/mL, Sigma, USA) for 24 h [27], followed by culturing in fresh medium for another 24 h. PMA-activated THP-1 cells were used for all *in vitro* infection studies. African green monkey kidney cell line Vero E6 (ATCC CCL158) was maintained in minimum essential medium (MEM, Gibco, USA) containing 2% heat-inactivated FBS, 1% penicillin-streptomycin solution (PS, Gibco, USA) and 1% L-Glutamine (LG, Gibco, USA) at 37°C with an atmosphere of 5% CO_2_.

DBV strain of DBV-ZJ/2021/01 (GenBank: OQ658514-OQ658516) was isolated from a 58-year-old SFTS patient in Zhejiang province of 2021 and was used for all tests. It was propagated in Vero E6 cells. Briefly, Vero cells were cultured in a 75 cm^2^ flasks at a density of 2 × 10^5^ cells and inoculated with 100 μL virus solution for 2 h. Then the Vero E6 monolayers were washed with phosphate buffer salt (PBS) and replenished with MEM (2% FBS, 1% LG and 1% PS). Virus supernatant was harvested after 5–7 days propagation (approximately 80%-90% of the cells have cytopathic effects). DBV titers were determined using a TCID_50_ assay with the Vero E6 cells as previously described [28]. Briefly, a 10-fold serial dilution of the culture was added to a monolayer of Vero E6 cells 100 μL per well followed by incubation for 7 days at 37°C with 5% CO_2_. After incubation, cytopathological changes above and below 50% virus dilution were recorded and then calculated using the Karber method [29]. Finally, TCID_50_ was converted to a plaque forming unit (PFU). PFU = 0.7 × TCID_50_ pfu/mL.

### Platelet-rich plasma

Platelet-rich plasma (PRP) was provided by Zhejiang provincial Blood Center and was isolated from the healthy human whole blood following a platelet isolation protocol as previously described [28]. In brief, peripheral blood samples from healthy volunteers were suspended in ACD solution (75 mM sodium citrate, 39 mM citric acid and 135 mM dextrose, pH 6.5) and then centrifuged at 150×*g* for 15 min at room temperature to obtain PRP. PRP was centrifuged at 750×*g* in the presence of ACD buffer for additional 15 min. The resulting pellet was suspended in pre-warmed Tyrode’s buffer (137 mM NaCl, 12 mM NaHCO_3_, 2 mM KCl, 0.34 mM Na_2_HPO_4_, 1 mM MgCl_2_, 5.5 mM glucose and 5 mM HEPES, pH 7.4) at the concentration of 2 × 10^11^ platelets/mL.

### Scanning electron microscopy (SEM) and optical microscopy

PMA-activated THP-1 macrophages (1 × 10^6^ cells per well) were plated on the coverslips in six-well plates (Corning, USA) and infected with DBV at a multiplicity of infection (MOI) of 0.1 or 1 pfu/cell. After 2 h absorption, the cells were gently washed with PBS and replenished with RPMI 1640 (2% heat-inactivated FBS) for 72 h incubation. Macrophages of positive control were performed with the treatment of 1 mg/mL LPS (Sigma, USA) for 4 h and 2 μM nigericin (Sigma, USA) for another 30 min. Untreated macrophages were served as negative control.

DBV-infected (MOI = 0.1 or 1) macrophages, nigericin/LPS-treated (Sigma, USA) macrophages, and untreated macrophages were subjected to morphology analysis by SEM and optical microscopy. For SEM analysis, all samples were washed with PBS and fixed with 2.5% (vol/vol) glutaraldehyde in PBS. The samples were postfixed in 0.5% reduced osmium, treated with 1% tannic acid, and dehydrated in gradient ethanol. After dehydration, the samples were applied with a small piece of adhesive tape to the apical surface and then coated with 75-A iridium. SEM images were obtained using a field emission scanning electron microscope (HITACHI, SU8010, Japan). The operating voltage was 2.5 kV.

### Cell viability analysis

PMA-activated THP-1 cells (1 × 10^4^ cells per well) were inoculated onto 96-well culture plates (Beyotime, Shanghai, China). The cells were untreated, or treated with LPS and nigericin, or infected with DBV at various MOIs (0.01, 0.1, 1) with or without the addition of platelets (5 × 10^5^ platelets per well) for 24 h, 48 h, 72 h and 96 h. Supernatants from 96-well culture plates were removed at indicated time points and added with 100 μL per well of CellTiter-Lumi Luminescence detection reagent (Beyotime, Shanghai, China) to promote cell lysis. The plates were incubated for 10 min at room temperature and cellular viability was detected by its luminescent signal intensity using a multi-function enzyme marker (PerkinElmer EnVision, Massachusetts, USA).

### Lactate dehydrogenase (LDH) release assay

LDH release reagent was added into the "maximum enzyme activity control hole of the sample" 1 h before the test. PMA-activated THP-1 macrophages (1 × 10^5^ cells per well) were then infected by DBV at MOIs of 0.01, 0.1, 1 in 96-well plates (Corning, USA) for 24 h, 48 h, 72 h and 96 h. Macrophages treated with LPS and nigericin or untreated were served as positive and negative control, respectively. Supernatants and cell lysates (120 μL) from each well were harvested at indicated time points after centrifugation at 400×*g* for 5 min, which were subjected to LDH release analysis. LDH release assays were performed using the LDH Cytotoxicity Assay Kit (PerkinElmer EnVision, Massachusetts, USA) according to the manufacturer’s instruction. The LDH activity in the culture supernatant was determined by reading the absorbance at OD_490_ nm and OD_600_ nm as a percentage of total LDH in the cell lysate.

### Immunofluorescence assay

To evaluate the macrophage-platelet interaction during DBV infection, 8 × 10^5^ PMA-activated THP-1 cells were seeded onto a confocal plate (Biosharp, Anhui, China) and infected by DBV (MOI = 1). Macrophages treated with LPS and nigericin or untreated were served as positive and negative controls, respectively. After 72 h incubation, macrophages were washed three times with precooled PBS, fixed with 1% paraformaldehyde for 5 min, and penetrated with 0.1% Triton-X for 10 min. After blocking with immunostaining blocking solution (Beyotime, Shanghai, China) for 10 min, samples were incubated overnight at 4°C with anti-tubulin antibody (1:200) (Abcam, Britain), anti-caspase-1 antibody (1:200) (Santa Cruz Biotechnology, USA) and anti-DBV-Gc antibody (1: 200) (Sangon Biotech, Shanghai, China). Samples were washed with PBS and incubated with donkey anti-rabbit IgG H&L (Alexa Fluor 568) pre-adsorbed (1:200) (Abcam, Britain), goat anti-mouse IgG H&L (Alexa Fluor 488) pre-adsorbed (1:200) (Abcam, Britain) and donkey anti-rat IgG H&L (Alexa Fluor 647) pre-adsorbed (1:200) (Abcam, Britain) for 1 h. The nuclei were then stained using the mounting medium with DAPI (Abcam, Britain). The images were obtained using a laser confocal microscope (ZEISS, LSM880, Germany).

### Real-time quantitative PCR

Total RNA from untreated, LPS/nigericin-treated, DBV-treated and DBV/platelets (5 × 10^7^ platelets per well)-treated macrophages (1 × 10^6^ cells per well) was collected at 24 h, 48 h, 72 h and 96 h post-exposure and was extracted using RNeasy Mini Kit (QIAGEN, Germany) and was homogenized using QIAshredder (QIAGEN, Germany) according to the manufacturer’s instructions, followed by reversion to cDNA using PrimeScrip RT Master Mix (TaKaRa, Japan). Each assay was performed in a 20 μL reaction volume containing 10 μL TB Green Premix Ex Taq II (Tli RNaseH Plus) (2×), 0.8 μL forward (10 μM), 0.8 μL reverse primers (10 μM), 0.4 μL ROX Reference Dye or Dye II (50×), 6 μL RNase-free water and 2 μL of cDNA template. The sequences of the primers used in this experiment were as follows: 5’-AACTCGCTATCCCTGTTGTCTAC-3’ and 5’-CCACCACTCGTCCAGCAAGAC-3’ for *GSDMD*; 5’-TCCAATAATGGACAAGTCAAGCC-3’ and 5’-GCTGTACCCCAGATTTTGTAGCA-3’ for *caspase-1*; 5’-TGTCCTGCGTGTTGAAAGATGAT-3’ and 5’-GCAGACTCAAATTCCAGCTTGTT-3’ for *IL-1β*; 5’-CTGTAGCCCATGTTGTAGCAAAC-3’ and 5’-TTGAAGAGGACCTGGGAGTAGAT-3’ for *TNF-α*; 5’-AACATGTGTGAAAGCAGCAAAGA-3’ and 5’-CTCTGGCTTGTTCCTCACTACTC-3’ for *IL-6*; 5’-GACGAAGAGAACTGGAGGAAGAT-3’ and 5’-TCAATCAGGAAGAGGGTGTTGTT-3’ for *ANPEP*; 5’-GCTGAACAGTGACAAATCCAACT-3’ and 5’-TGCTCAAACACATTAGGCACAAT-3’ for *TCAATCAGGAAGAGGGTGTTGTT*; 5’-CGAAGTCATAGCCACACTCAAGA-3’ and 5’-CTGCAGGAAGTGTCAATGATACG-3’ for *CXCL-3*; 5’-GAAGGTCGGAGTCAACGGATTT-3’ and 5’-TTGATGACAAGCTTCCCGTTCTC-3’ for *GAPDH* (Tsingke Biotechnology, Beijing, China). Amplifications were performed on a 7500 Fast Real-Time PCR system (Applied Biosystems, Waltham, MA, United States) using the following conditions: 95°C for 30 s, followed by 40 cycles of 95°C for 5 s and 60°C for 34 s. Data were analyzed by the 2^-ΔΔCt^ method.

### Enzyme-linked immunosorbent assay (ELISA)

To examine the secretion of IL-1β, TNF-α, IL-6, IL-8, IL-10 and IL-12 from macrophages, culture supernatants from untreated, LPS/nigericin-treated, DBV-treated and DBV/platelets (5 × 10^7^ platelets per well)-treated macrophages (1 × 10^6^ cells per well) were harvested at 24 h, 48 h, 72 h and 96 h post-exposure. All samples were centrifuged at 12000 rpm for 5 min before collection and stored at -80°C for further cytokine quantitation. The concentrations of mature human IL-1β, TNF-α IL-6, IL-8, IL-10 and IL-12 were measured according to the manufacturer’s protocol using specific ELISA Kits (Solarbio, Beijing, China), respectively. The absorbance was measured following the addition of 100 μL stop solution (2N H_2_SO_4_) at OD_450_ nm by a multi-function enzyme marker (PerkinElmer EnVision, Massachusetts, USA).

### Western blot

To determine the protein expression levels of caspase-1, GSDMD, IL-1β and β-actin in human macrophages, total protein from untreated, LPS/nigericin-treated, DBV-treated and DBV/platelets (5 × 10^7^ platelets per well)-treated macrophages (1 × 10^6^ cells per well) was collected at 24 h, 48 h, 72 h and 96 h post-exposure and was lysed by RIPA buffer (Solarbio, Beijing, China) containing with a mixture of protease inhibitors (Solarbio, Beijing, China). The concentration of protein was determined by a BCA protein assay kit (Beyotime, Shanghai, China) and mixed with SDS loading buffer (Beyotime, Shanghai, China). Samples were electrophoresed in SDS-polyacrylamide gel electrophoresis gel and transferred onto polyvinylidene fluoride membranes (Millipore, USA). The membranes were blocked with 5% nonfat milk in TBST buffer (50 mM Tris/HCl, pH 7.4, 150 mM NaCl and 0.1% Tween-20) for 1 h and incubated with the indicated primary antibodies (1: 1000) including gasdermin D (E8G3F) rabbit mAb 97558, cleaved gasdermin D (Asp275, E7H9G) rabbit mAb 36425, caspase-1 (D7F10) rabbit mAb 3866, cleaved caspase-1 (Asp297, D57A2) rabbit mAb 4199, IL-1β (D3U3E) rabbit mAb 12703, cleaved IL-1β (Asp116, 3A3Z) rabbit mAb 83186 (Cell Signaling Technology, Germany) or NLRP3 rabbit pAb (ZENBIO, Chengdu, China) at 4°C overnight. Afterward, the membranes were incubated with 1: 10000 anti-rabbit HRP-conjugated secondary antibody or anti-mouse HRP-conjugated secondary antibody (Abcam, Britain) for 1 h at room temperature. The signals were visualized with an enhanced chemiluminescence substrate (Biosharp, Anhui, China) using a chemiluminescence imaging system (Tanon, Shanghai, China). Image J Fiji software (National Institutes of Health, Bethesda, MD, USA) was used to quantify the protein abundance.

### RNA sequencing and analysis

DBV-infected macrophages (1 × 10^6^ cells per well) were co-incubated with (platelet added, 5 × 10^7^ platelets per well, cohort 1) or without platelets (no platelet, cohort 2) for 72 h and then washed macrophages were collected to extract total RNA using the RNeasy Mini kit (QIAGEN, Germany). Extracted total RNA of each sample was quantified by NanoDrop 2000 (Thermo Fisher Scientific, Waltham, MA) spectrophotometer, followed by verification using agarose gel electrophoresis. Samples with RNA integrity number ≥ 8.0 were selected and sent to professional sequence company (Shanghai Tanpu Biotechnology Co., Ltd) for sequencing library preparation. The quadruplicate samples of all assays were constructed an independent library, and the size distribution was analyzed using Agilent BioAnalyzer. Sequencing libraries for cohorts 1 and 2 were barcoded and prepared using TruSeq V2 with oligo-dT selection (Illumina, San Diego CA, USA). RNA sequencing was performed on an Illumina Novaseq 6000 platform to generate 2 x 150 bp paired-end reads. Sequencing and library construction were performed by technical staff at Shanghai Tanpu Biotechnology Co., Ltd.

QC quality control of original RNA-sequencing data was performed on all samples using Fast QC (v0.11.9) software. FASTQ reads (≥ 75 bp length) were obtained by removing the junction sequences using Fastp (v0.20.1) software [30], and were aligned with Novocraft’s Novo alignment program (Novocraft Technologies, Selangor, Malaysia) to analyze of expression variation. The DeSeq2 analysis package was used to normalize read counts for each cohort and assign the reads to composite transcripts (1 per gene) [31]. RSEM (v1.3.3) was used to evaluate the expression of all genes or transcripts in each sample. The edge R (v3.28.1) package of the R language was then used to standardize the expression amount of the obtained gene or transcript by using trimmed mean of M values and count per million. The expression amount of these genes was subsequently subjected to principal component analysis (PCA) and cluster analysis. Differential expression analysis used the overmatched Poisson model to calculate read counts, and Empirical Bayes corrected the dispersion of all gene expression levels [32]. The screening criteria for significantly differentially expressed genes are adjusted *P* value < 0.05 and |FC| ≥ 2 (FC = the fold change of expressions).

### Statistical analysis

All experimental data were represented as means ± standard deviation. Statistical analysis was carried out by one-way ANOVA. IBM SPSS Statistics 28.0 and GraphPad Prism 9 were used for all statistical analysis and charts generation. *P* value < 0.05 was considered statistically significant.

## Result

### DBV induces pyroptotic cell death and classical morphology in human macrophages

**Fig 1:**
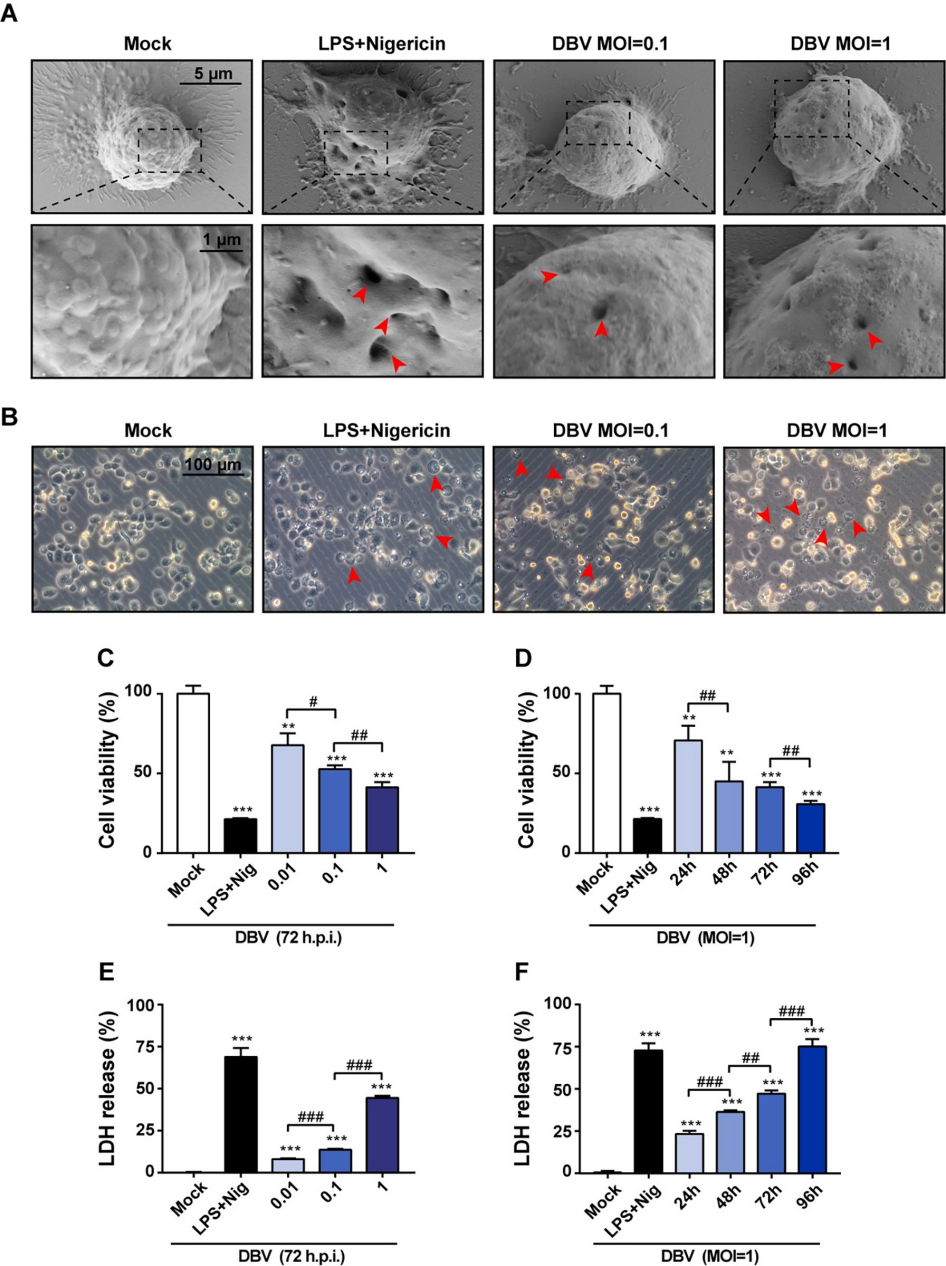
DBV-infected macrophages exhibit pyroptosis-specific cell morphology and cell death. PMA-activated THP-1 cells were incubated on coverslips in six-well plates with different concentrations of DBV (MOI = 0.1, 1), LPS and nigericin, or culture medium. After 72 h of incubation, unbound cells were washed out, and images were obtained by **(A)** scanning electron microscopy and **(B)** inverted microscopy analysis at a magnification of ×200. The red arrow indicates classical morphological features of pyroptosis. Representative images of three replicates per macrophage group were shown. PMA-activated THP-1 cells were treated with different doses of DBV (MOI = 0.01, 0.1, 1) for 72 h incubation, or infected with DBV at MOI of 1 for 24 h, 48 h, 72 h and 96 h incubation, or treated with LPS and nigericin, or were mock infected. **(C and D)** After incubation at indicated time points, cell viability was detected by the CCK-8 kit. **(E and F)** Supernatants from different coculture conditions were collected, and cell death was determined using an LDH release assay. Data were mean values ± SD derived from the samples collected in triplicate. *P<0.05, **P<0.01, ***P<0.001 compared with mock; ^#^*P*<0.05, ^##^*P*<0.01, ^###^*P*<0.001 compared between different treatments, by one-way ANOVA test.

To determine the potential presence of pyroptosis in macrophages during DBV infection, we first investigated the morphogenesis of DBV-infected THP-1 macrophages by SEM and optical microscopy. Hole-like structures indicative of pyroptosis were found in human macrophages infected with DBV or treated with LPS and nigericin combination compared to the intact cell membrane in the negative control group **(Fig 1A)**. Optical microscope observation at the same time confirmed DBV-infected macrophages exhibited typical cytopathic effects (CPE) in pyroptosis-mediated cell death, including cell hypertrophy and transparent cytoplasm **(Fig 1B)**. Pyroptosis-like CPE was consistently displayed in different MOIs of DBV-treated macrophages and the magnitude of the increase depended on the infectious dose as well as the infection time **(S1 Fig)**. Additionally, immunofluorescence staining using specific DBV Gc antibody and cleaved caspase-1 observed that DBV was widely distributed within caspase-1^+^ macrophagic niches and accumulated around the nucleus at 72 h post-infection, which might facilitate pyroptotic cell death of macrophages **(S2 Fig)**.

To further illustrate the biological alterations of macrophages in response to DBV infection, we examined cell viability and LDH production of macrophages. A high percentage of the macrophages that infected with DBV were exhausted and their cellular viability decreased gradually with the increase of DBV MOIs **(Fig 1C)**. Meanwhile, the cell viability reduced markedly as time progressed **(Fig 1D)**. DBV invasion also resulted in the release of LDH, and the rate of LDH secretion increased in a DBV-dose and time-dependent manner **(Fig 1E and 1F)**.

### Induction of pyroptosis is associated with activated NLRP3 inflammasome and increased IL-1β

**Fig 2:**
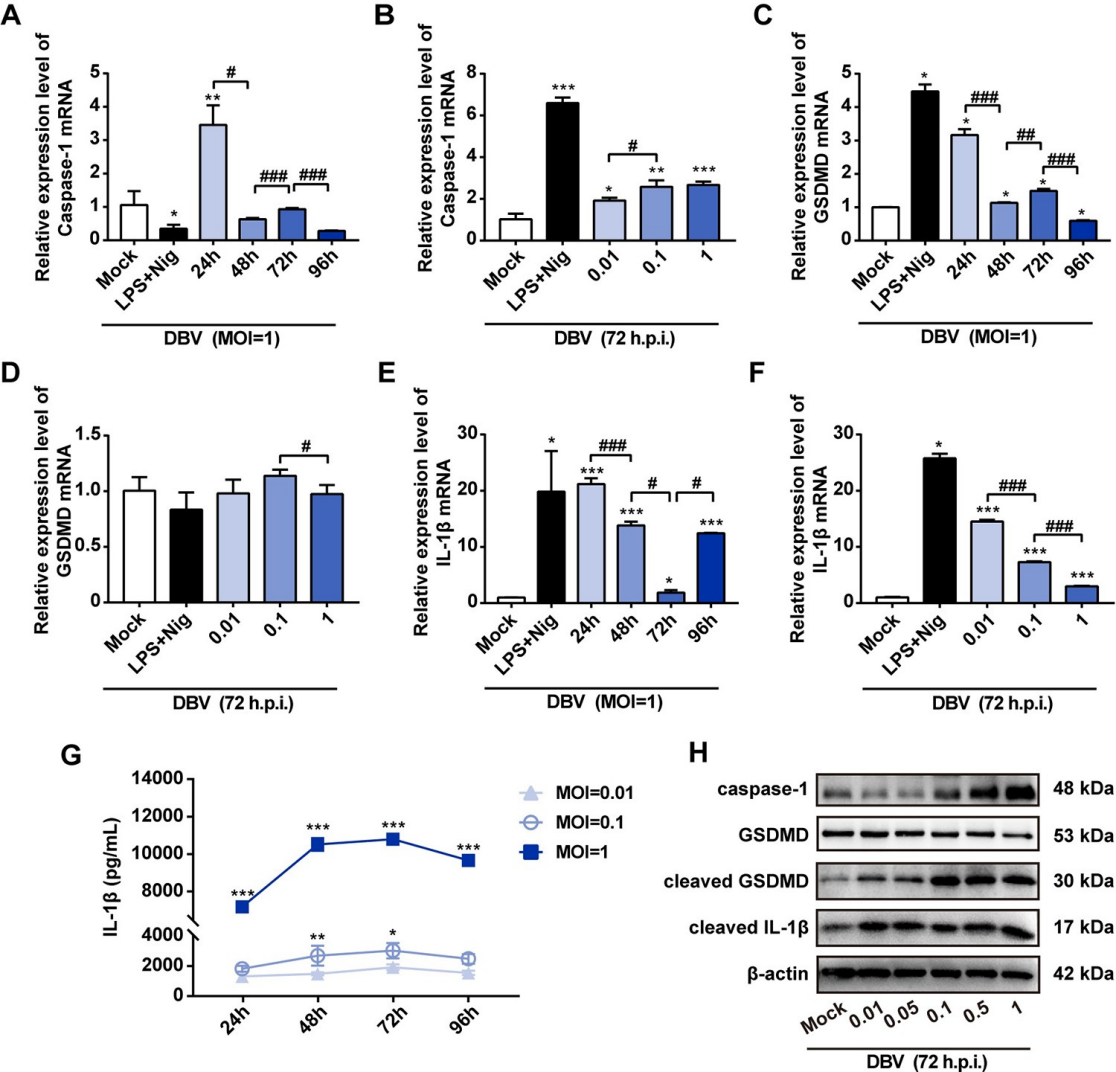
DBV infection induces pyroptosis characterized by caspase-1 and GSDMD activation and IL-1β secretion. **(A and B)** Relative expression levels of caspase-1 mRNA in macrophages were quantified by RT-qPCR. **(C and D)** Relative expression levels of GSDMD mRNA in macrophages were quantified by RT-qPCR. **(E and F)** Relative expression levels of IL-1β mRNA in macrophages were quantified by qPCR. A to F: Data were mean values ± SD derived from the samples collected in triplicate. *P<0.05, **P<0.01, ***P<0.001 compared with mock; ^#^*P*<0.05, ^##^*P*<0.01, ^###^*P*<0.001 compared between different treatment conditions, by one-way ANOVA test. **(G)** Macrophages were infected with DBV at different MOIs (0.01, 0.1, 1). At indicated time points, supernatants from different coculture conditions were collected, and IL-1β levels were measured by ELISA kit. Data were mean values ± SD derived from the samples collected in triplicate. *P<0.05, **P<0.01, ***P<0.001 compared with MOI = 0.01, by one-way ANOVA test. **(H)** Examination of the proteolytic cleavage of caspase-1, GSDMD and IL-1β in human macrophages with DBV or mock infection using western blot analysis (72 h.p.i). β-actin was probed as a loading control.

Execution of pyroptosis is an inflammasome-mediated process. Once activated, the NLRP3 inflammasome triggers the auto-cleavage of caspase-1, an effective factor that subsequently facilitates the proteolytic processing of gasdermin D (GSDMD) and induces highly inflammatory form of cell death known as pyroptosis [33,34]. To further confirm the induction of pyroptosis in macrophages, we assessed the downstream effector of caspase-1 in macrophages by RT-qPCR, ELISA and western blot. The result demonstrated that the expression of caspase-1 mRNA gradually decreased over time post-infection, while no significant association was observed between DBV dose and caspase-1 activation **(Fig 2A and 2B)**. We also confirmed that DBV-triggered pyroptosis was in a GSDMD-dependent process, as evidenced by the increase of GSDMD and cleaved-GSDMD protein expression **(Fig 2C and 2H)**. The expression of GSDMD mRNA reached its maximum at 24 h post-infection, while no significant correlation between DBV dose and the expression level was observed **(Fig 2D)**.

The release of proinflammatory cytokine IL-1β is a hallmark characteristic of pyroptosis, leading to inflammatory cell lysis [35]. We next examined the effects of DBV infection on the expression of IL-1β mRNA in macrophages. RT-qPCR analysis revealed that DBV infection rapidly escalated the expression of IL-1β mRNA at 24h, and its mRNA expression level was negatively correlated with DBV concentration **(Fig 2E and 2F)**. To confirm whether induction of pyroptosis in macrophages is associated with cytokine secretion, cytokine levels (IL-1β, IL-6, IL-8, IL-10, IL-12 and TNF-α) in the supernatant of DBV-infected (MOI = 0.01, 0.1, 1) macrophages were assessed by ELISA. Notably, IL-1β levels significantly increased in the supernatants of high DBV concentration-treated macrophages (MOI = 1) and reached its peak at 72 h post-infection, in contrast to the macrophages treated with low DBV doses (MOI = 0.01 or 0.1) showing minimal production of IL-1β **(Fig 2G)**. A modest but significant release of other inflammatory cytokines including TNF-α, IL-6, IL-8, IL-10 and IL-12 were also observed after 72 h post-treatment **(S3 Fig)**. In addition, the trends in cleaved IL-1β protein were similar to those observed for IL-1β mRNA expression **(Fig 2H)**. These results together suggested that DBV-mediated pyroptosis executed in a caspase-1 and GSDMD-dependent pathway and induced IL-1β release in macrophages.

### The human macrophage transcriptome is altered in the presence of platelet during DBV infection

Platelets had a protective role in inflammasome activation [36,37]. Since thrombocytopenia is the hallmark clinical symptom in lethal SFTS, we speculated that platelets might influence DBV-induced pyroptosis in macrophages. To determine whether the macrophage transcriptome is altered in the presence of platelet during DBV infection, we performed RNA-seq on total RNA isolated from DBV-infected macrophages at 72 h post-exposure with or without the addition of platelets.

**Fig 3:**
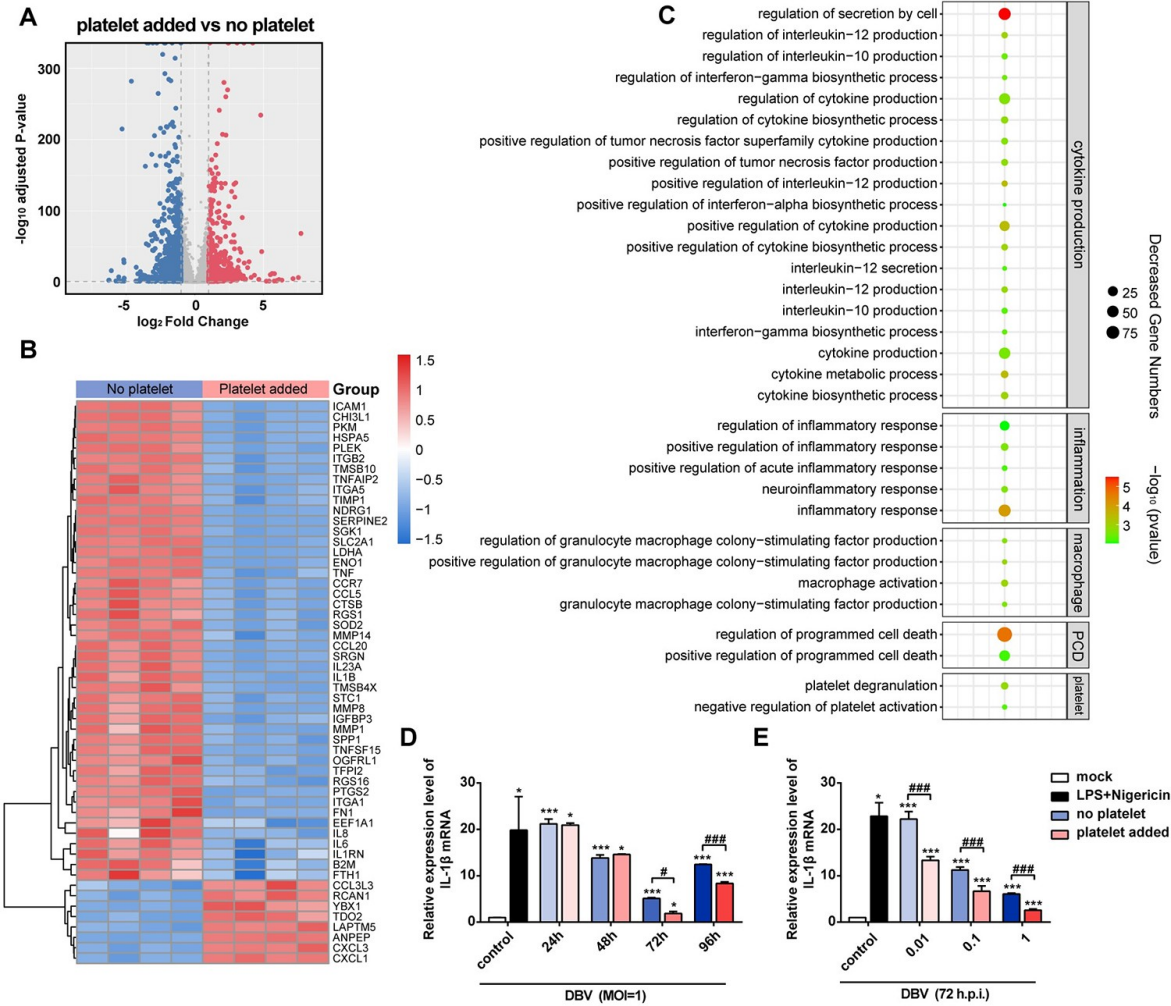
Platelet addition altered the transcriptome of DVB-infected macrophages. Macrophages were infected with DBV at MOI of 1 for 72h as a “no platelet” group (n = 4), or infected with DBV at MOI of 1 with platelets for 72h as a “platelet added” group (n = 4). **(A)** Volcano plot analysis of mRNAs differentially expressed in response to the “platelet added” group vs “no platelet” group. Red dots indicate up-regulated genes and blue dots indicate down-regulated genes. **(B)** Heatmap of significantly differentially expressed macrophage transcripts from the “no platelet” group and “platelet added” group. Red represents enhanced relative expression, and blue represents reduced relative expression. **(C)** GO enrichment analysis of differentially expressed mRNAs for no-platelets and platelet-added macrophages. **(D and E)** IL-1β mRNA in macrophages was quantified by RT-qPCR. Data were mean values ± SD derived from the samples collected in triplicate. *P<0.05, **P<0.01, ***P<0.001 compared with mock; ^#^*P*<0.05, ^##^*P*<0.01, ^###^*P*<0.001 compared between different treatment conditions, by one-way ANOVA test.

As shown in **Fig 3A**, 619 genes were up-regulated and 904 genes were down-regulated in platelet-added macrophages, suggesting systemic alterations in macrophagic RNA expression. The most down-regulated gene in the “platelet added” group was *IL1B*, which encodes protein IL-1β **(Fig 3B)**. Other inflammatory cytokine genes including *IL8*, *IL6*, *IL23A*, *IL1RN*, *TNFSF15* and *TNF* were also significantly down-regulated in platelet-added macrophages. In addition to cytokine-related genes, clotting genes (*SERPINE2*, *TFPI2* and *FN1*) and protease genes that are associated with macrophage activation (*MMP1*, *MMP8* and *MMP14*) were down-regulated in the “platelet added” group as well. Meanwhile, several genes such as *CXCL1* and *CXCL3* (chemotactic agents for neutrophils), *RCAN1* (suppression neurocalcin signaling pathway) and *ANPEP* encoding blood vessel production were up-regulated in “platelet added” samples.

Gene ontology analysis was further performed to investigate the function of differentially expressed mRNAs. As expected, the results revealed that the most strongly regulated biological groups are related to “cytokine production”, inflammatory response”, “macrophage activity”, “programmed cell death” and “platelet activity” **(Fig 3C)**. To assess the relevant changes of pyroptosis–related genes, we used RT-qPCR to measure the expression level of IL-1β mRNA in macrophages. Consistent with the transcriptome findings, platelet addition reversed the expression level of IL-1β mRNA in macrophages and the expression of IL-1β mRNA was significantly down-regulated in platelet-added macrophages at 72 h and 96 h **(Fig 3D)**. Likewise, this phenomenon was observed in all macrophages with different DBV dose treatments **(Fig 3E)**. Accordingly, RT-qPCR analysis confirmed the same alterations in mRNA expression of other up and down-regulated genes associated with different biological functions **(S4 Fig)**. Taken together, our data suggested that the macrophage transcriptome was altered in the presence of platelets during DBV infection.

### Platelets attenuated DBV-mediated pyroptosis and inflammatory responses in macrophages

**Fig 4:**
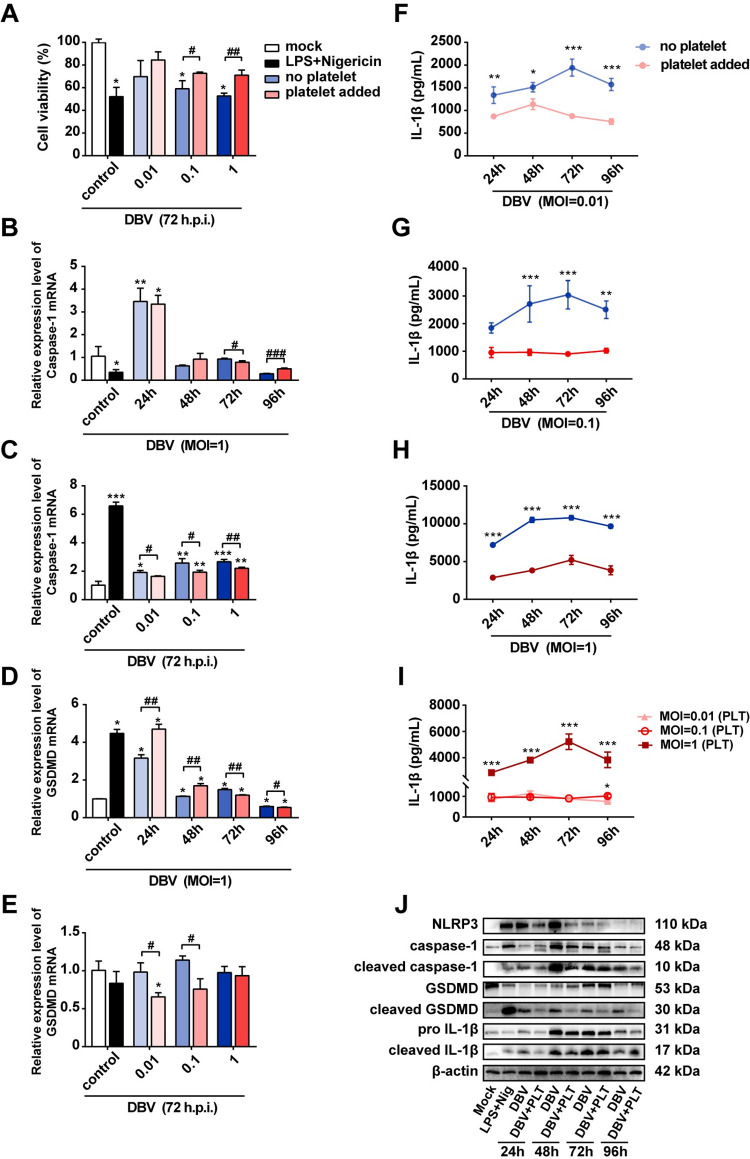
DBV-mediated macrophage pyroptosis and inflammatory responses are attenuated by platelets. Macrophages were mock infected, or treated with LPS and nigericin, or infected with DBV at MOIs of 0.01, 0.1, 1, or co-incubated with platelets and DBV at MOIs of 0.01, 0.1, 1. **(A)** After incubation at required time points, cell viability was measured by the CCK-8 kit. **(B, C)** caspase-1 mRNA in macrophages from different coculture conditions were quantified by RT-qPCR. **(D, E)** GSDMD mRNA in macrophages from different coculture conditions was quantified by RT-qPCR. A to E: Data were mean values ± SD derived from the samples collected in triplicate. *P<0.05, **P<0.01, ***P<0.001 compared with mock; ^#^*P*<0.05, ^##^*P*<0.01, ^###^*P*<0.001 compared between different treatment conditions, by one-way ANOVA test. **(F-I)** IL-1β levels in supernatants were determined by ELISA kit. *P<0.05, **P<0.01, ***P<0.001 compared with MOI = 0.01, by one-way ANOVA test. **(J)** NLRP3, caspase-1, GSDMD, pro IL-1β as well as their cleaved proteins in the cell lysates were determined by western blot. β-actin was probed as a loading control.

Platelets are crucial for optimal inflammasome activation and optimal production of IL-1 cytokines in macrophages [36,38]. Platelets have shown a protective role in sepsis through the down regulation of macrophage-dependent proinflammatory cytokines (TNF-α and IL-6) [37]. In SFTS, platelets have reported to enhance the transportation of DBV to macrophages for clearance [14,28]. Thus, we next evaluated the platelet effects in macrophagic pyroptosis and inflammatory responses during DBV infection. DBV-infected macrophages were incubated with or without the addition of platelets *in vitro* for multiple time points. As shown in **Fig 4A**, the inhibitory effect of DBV (MOI = 1 or 0.1) on cell viability was reversed in the presence of platelets after 72 h exposure. Platelets also suppressed the mRNA expression levels of pyroptosis-related genes (GSDMD and caspase-1) and displayed distinct modulation outcomes over the time of infection **(Fig 4B–4E)**. For example, platelets significantly inhibited the expression of caspase-1 mRNA in macrophages at all DBV infectious doses at 72 h post-infection, while only reduced the expression of GSDMD when the DBV MOI was 0.1 or 0.0.1.

Further, we observed a significant decrease in the release of IL-1β in the supernatant of DBV (PLT)-coinfected macrophages compared to the DBV (only)-infected macrophages on almost all exposure time points **(Fig 4F–4H)**. Despite the fact that IL-1β secretion was reduced by platelets, DBV still triggered IL-1β release in a dose-dependent manner **(Fig 4I)**. Accordingly, the production of other inflammatory cytokines (TNF-α, IL-6 and IL-8) in the DBV (PLT) group was also significantly lower than that in DBV (only) group at all time points **(S5 Fig)**.

Consistent with transcriptome findings, western blot results revealed that platelets inhibited pro-IL-1β cleavage to cleaved IL-1β. Platelets also had a significant impact on the expression and cleavage of caspase-1, GSDMD and IL-1β. As shown in **Fig 4J**, platelets boosted the expressions of NLRP3, caspase-1 and GSDMD, whereas the expressions of cleaved GSDMD and cleaved caspase-1 were inhibited by platelets in DBV-infected macrophages. These results together demonstrated that platelets inhibited macrophagic pyroptosis and inflammatory responses during DBV infection.

## Discussion

Macrophages are one of the principal targets for DBV replication in fatal SFTS cases [21]. Previous studies have demonstrated that DBV infection did not activate apoptotic cell death in macrophages [13,21], but triggered the activation of NLRP3 inflammasome and extracellular release of IL-1β [16,39], suggesting proinflammatory cell death might be involved in DBV-infected macrophages. However, the precise mechanisms underlying the demise of macrophages during DBV infection remain poorly explored. Here we showed that DBV can induce pyroptosis and inflammatory responses in macrophages. Pyroptosis is recognized as its own entity of programmed cell death that induces strong inflammatory responses to help host defense against microbial infection [40]. Many viral infections were reported to trigger pyroptosis in host cells, such as human bocavirus 1 [41], influenza A virus [42] and dengue virus [43]. Overwhelmed pyroptosis resulted in excessive tissue inflammation, multi-organ failure and poor prognosis, and these symptoms have been found in severe SFTS patients [44].

**Fig 5:**
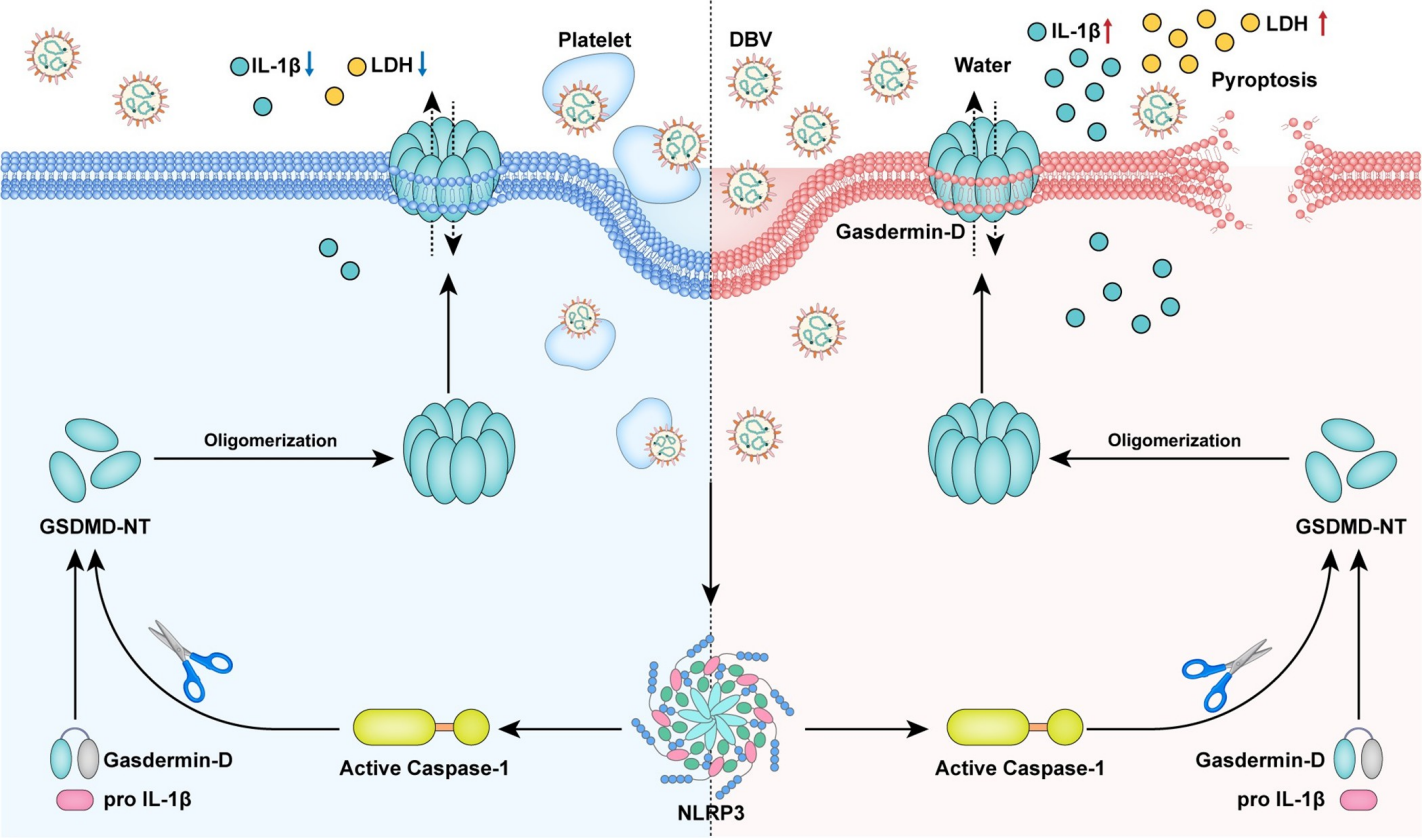
Model depicting DBV-mediated pyroptosis and inflammatory responses in macrophages. In SFTS, DBV virions are phagocytosed by human macrophages in the circulation. DBV takes advantage of human macrophages for its own replication but triggers the activation of inflammasome NLRP3. NLRP3 inflammasome activation then induces the auto-cleavage of caspase-1. Once assembled, caspase-1 mediates the proteolytic process of GSDMD and IL-1β, triggering pyroptosis to facilitate host defense (right). On the contrary, in the presence of platelets, DBV-infected macrophages show suppressed activation of caspase-1 and GSDMD and reduced inflammatory response related to IL-1β in the same process, protecting human macrophages from pyroptosis (left).

Consistent with clinical observations, our study confirmed that infected macrophages displayed classical morphological hallmarks of pyroptosis including non-selective pore-forming plasma membrane rupture, cytoplasm transparency and cell body expansion. The prototypical form of pyroptosis requires the involvement of caspase-1 (proinflammatory caspase) and pore-forming protein GSDMD [19]. Cleaved GSDMD served as an executioner of pyroptosis, which consists of an N-terminal domain, can oligomerize and form holes in the cell membrane [35]. These formed pores cause the rupture of the plasma membrane, but at the same time facilitate the release of proinflammatory mediators including IL-1β and IL-18 [34,45]. In agreement with the pyroptotic pathway, the expression of pyroptosis-related mRNA (GSDMD, caspase-1 and IL-1β) and the cleavage of pyroptosis-related protein (GSDMD, caspase-1 and IL-1β) were detected in macrophages. These results supported the notion that DBV infection induced pyroptosis in macrophages **(Fig 5)**.

Among severe SFTS patients, the occurrence of detrimental cytokine storm was heralded by high levels of serum proinflammatory cytokines [10]. IL-1β is a potent proinflammatory cytokine that is essential for the host response and resistance to pathogens [46]. A unique pattern of IL-1β elevation in fatal SFTS cases was previously noted [24] and was reaffirmed by the present study, as the detectable release of IL-1β rapidly started 24h post-infection and then escalated, especially at 72h and DBV MOI of 1. The landscape of IL-1β release was also concomitant with the replication of DBV in human macrophages [28]. Thus, our study supports the hypothesis that IL-1β plays an important role in the SFTS progress [47]. Released IL-1β, TNF-α and other inflammatory signals may attract more macrophages to die in a pyroptotic pathway and create a vicious pathogenic cycle, contributing to the disease severity in lethal DBV infections [48,49]. Although pyroptotic cell death is an antiviral host defense to curtail survival and replication of DBV, this programmed cell death acts as a double-edged sword and may lead to disease severity [50]. Previous study has reported pyroptosis as a primary cause of anthrax-lethal toxin–induced lung injury [51], resulting in the depletion of hematopoietic progenitor cells [52]. In addition, pyroptosis has been found to drive CD4^+^ T-cell depletion and chronic inflammation in HIV-1 infection [53]. Pyroptosis cell death and increased inflammatory cytokines may have similar functions in fatal SFTS to cause dysregulated host immunity and poor clinical outcomes [54]. Therefore, development of anti-pyroptotic therapies or caspase-1 inhibitors as pyroptosis-targeted interventional strategies might be promising in the future for the treatment of SFTS but warrants rigorous investigation.

Thrombocytopenia is a hallmark clinical manifestation in SFTS [55]. Previous animal model has revealed phagocytized platelet-DBV complex concentrated in murine spleen and replicated in large quantities [14]. In this study, we further investigated the effect of platelets on macrophagic pyroptosis. Notably, platelets significantly inhibited DBV-mediated pyroptosis in macrophages, as evidenced by reversed secretion of IL-1β and suppressed expression of N-GSDMD and pyroptosis-related mRNA, protecting hosts from IL-1-driven inflammation. However, the mechanism of platelets modulating pyroptosis and inflammatory response has not been fully understood and is likely to be complex. Previous studies have shown that platelet factor 4 (PF4), an antiangiogenic chemokine produced by platelets, increased p38 MAPK activation and downregulated STAT-2-IRF-9 signaling to decrease the synthesis and secretion of IL-1β and other inflammatory cytokines [56]. It is plausible that platelets affect the secretion of IL-1β and mediated macrophagic pyroptosis through the secretion of PF4 [57]. Further investigation into the medical significance of platelet-released mediators may provide a better understanding of the mechanisms of activity.

Platelets have been increasingly recognized for their functions of explicit host immune effector cells [58]. Platelet-mediated immune response has become a landmark event in many viral infections, including human immunodeficiency virus, hepatitis C virus, H_1_N_1_ influenza, Ebola and Dengue viruses [59–63]. Previous studies demonstrated a unique participation of platelets in the inflammasome output of innate immune cells [36,56]. Our findings propose a novel role of platelets in modulating immune responses in SFTS, while it remains controversial if platelet activities are beneficial for the host or for the DBV. Platelet addition reduced cellular death but also potentiated viral replication in macrophages [28], which may prolong the time for potential infection of macrophages by DBV. Disseminated progeny DBV in the circulation may aggravate platelet-virus interplay, thereby contributing to thrombocytopenia in SFTS. Our recent work demonstrated that platelets can rapidly internalize DBV through glycoprotein VI receptor and cause platelet depletion [28]; however, it is unclear if this process initiates programmed cell death in platelets to promote thrombocytopenia [64]. Thus, rational regulation of the impact of platelets on host immunity might be another key to SFTS treatment.

In summary, our study provides the first observation that DBV can induce pyroptosis and initiate the production of proinflammatory cytokines in macrophages. Although many innate immune pathways are engaged against DBV infection, we highlight the importance of the pyroptotic cell death pathway in human macrophages which removes the intracellular replicative niche for the viruses, making them susceptible to clearance. Additionally, we uncover a unique function of platelets that hamper the inflammasome activation in macrophages. Nevertheless, our present data was limited to *in vitro* experiments, whether and in what context pyroptosis impedes DBV infection in humans warrants further validation. Also, it would be interesting to decipher the complex roles of platelets in the host-DBV interaction in future research. Particularly, whether and how programmed cell death occurred in DBV-infected platelets requires rigorous investigation. Such investigation could be helpful to develop platelet treatment options for DBV-related diseases.

## Supporting information

S1 Fig DBV-infected macrophages exhibit pyroptosis-specific cell morphology. **(A)** Macrophages were treated with DBV (MOI = 1), LPS and nigericin, or were mock treated and incubated for 24 h, 48 h, 72 h or 96 h; **(B)** macrophages were infected with DBV at MOIs of 0.01, 0.1, 1. After incubation for 72 h, cell cultures from different treatments were subjected to inverted microscopy analysis at a magnification of ×200.
(TIF)

S2 Fig Co-visualization for pyroptotic protein in DBV-infected macrophages. Macrophages were infected with DBV (MOI = 1), or were mock infected on the coverslip in confocal dishes for 72 h, followed by washing and fixation. Macrophages were stained for tubulin (red), nuclei (blue) and caspase-1 (violet); adherent DBV virions were stained for Gc glycoproteins (green). Samples were subjected to laser confocal microscope at a magnification of ×630.
(TIF)

S3 Fig DBV-induced pyroptosis leads to cytokines secretion in macrophages. Macrophages were infected by DBV (MOI = 1) or were mock infected for 72 h. Then the supernatants were collected to measure **(A)** TNF-α, **(B)** IL-6, **(C)** IL-8, **(D)** IL-10 and **(E)** IL-12 production by ELISA. Data were mean values ± SD derived from the samples collected in triplicate. *P<0.05, **P<0.01, ***P<0.001, by two-tailed student *t* test.
(TIF)

S4 Fig Platelets altered mRNA expression of the genes involving macrophage activity and cytokine production in DBV-infected macrophages. DBV-infected (MOI = 1) macrophages were co-incubated with or without platelets for 72 h. The mRNA expression levels of **(A)** TNF-α, **(B)** IL-6, **(C)** ANPEP, **(D)** CXCL-1 and **(E)** CXCL-3 were detected by RT-qPCR and analyzed by 2^ΔΔCt^ method. Data were mean values ± SD derived from the samples collected in triplicate. *P<0.05, **P<0.01, ***P<0.001, *NS* = no statistical difference, by two-tailed student *t* test.
(TIF)

S5 Fig Cytokines secretion DBV mediated macrophage are attenuated by platelets. DBV-infected (MOI = 1) macrophages were co-incubated with or without platelets for 72 h. The cytokines levels of **(A)** TNF-α, **(B)** IL-6, **(C)** IL-8, **(D)** IL-10 and **(E)** IL-12 in the supernatants were analyzed by ELISA tests. Data were mean values ± SD derived from the samples collected in triplicate. *P<0.05, **P<0.01, ***P<0.001, *NS* = no statistical difference, by two-tailed student *t* test.
(TIF)
