# Supplementary figures and images for: Dabie bandavirus infection induces macrophagic pyroptosis and this process is attenuated by platelets
Source: PLoS Negl Trop Dis. 2023 Jul 24;17(7):e0011488. doi: 10.1371/journal.pntd.0011488 (PMC10399884; doi:10.1371/journal.pntd.0011488)

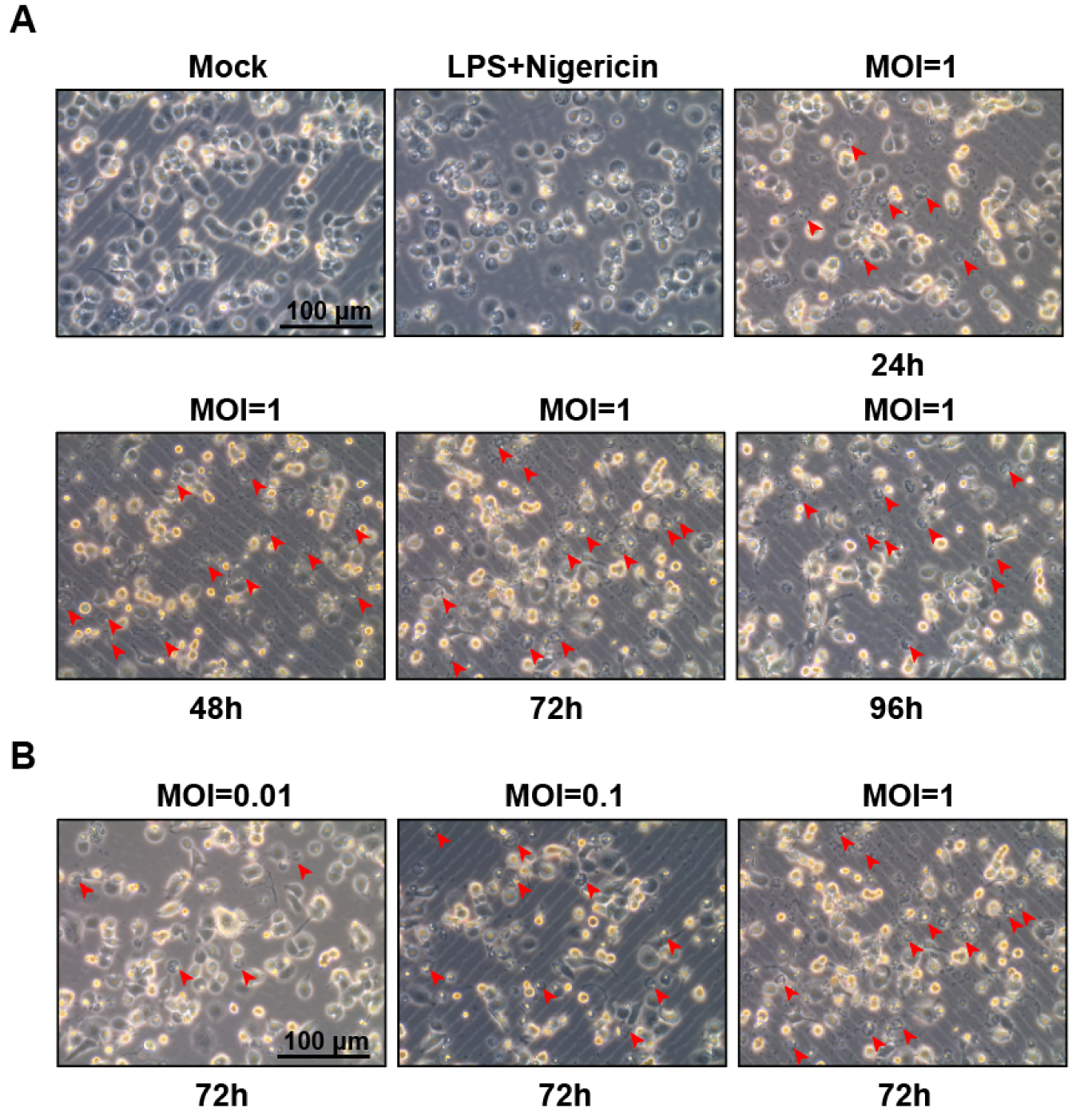

Supplement: S1 Fig — (A) Macrophages were treated with DBV (MOI = 1), LPS and nigericin, or were mock treated and incubated for 24 h, 48 h, 72 h or 96 h; (B) macrophages were infected with DBV at MOIs of 0.01, 0.1, 1. After incubation for 72 h, cell cultures from different treatments were subjected to inverted microscopy analysis at a magnification of ×200. (TIF) [file pntd.0011488.s001.tif]

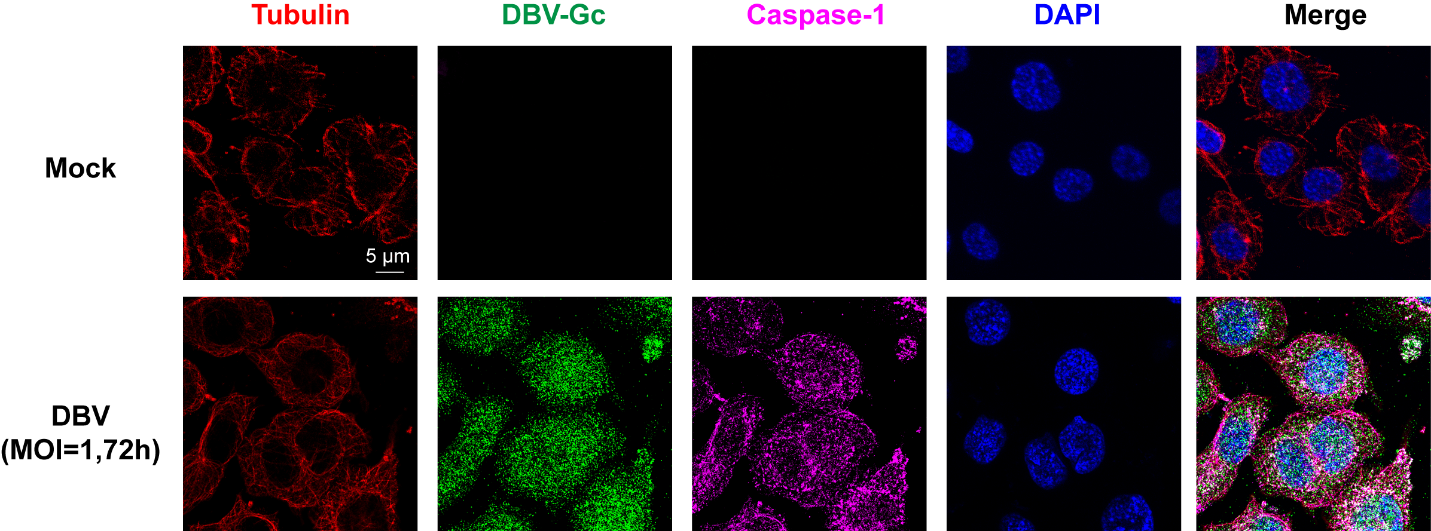

Supplement: S2 Fig — Macrophages were infected with DBV (MOI = 1), or were mock infected on the coverslip in confocal dishes for 72 h, followed by washing and fixation. Macrophages were stained for tubulin (red), nuclei (blue) and caspase-1 (violet); adherent DBV virions were stained for Gc glycoproteins (green). Samples were subjected to laser confocal microscope at a magnification of ×630. (TIF) [file pntd.0011488.s002.tif]

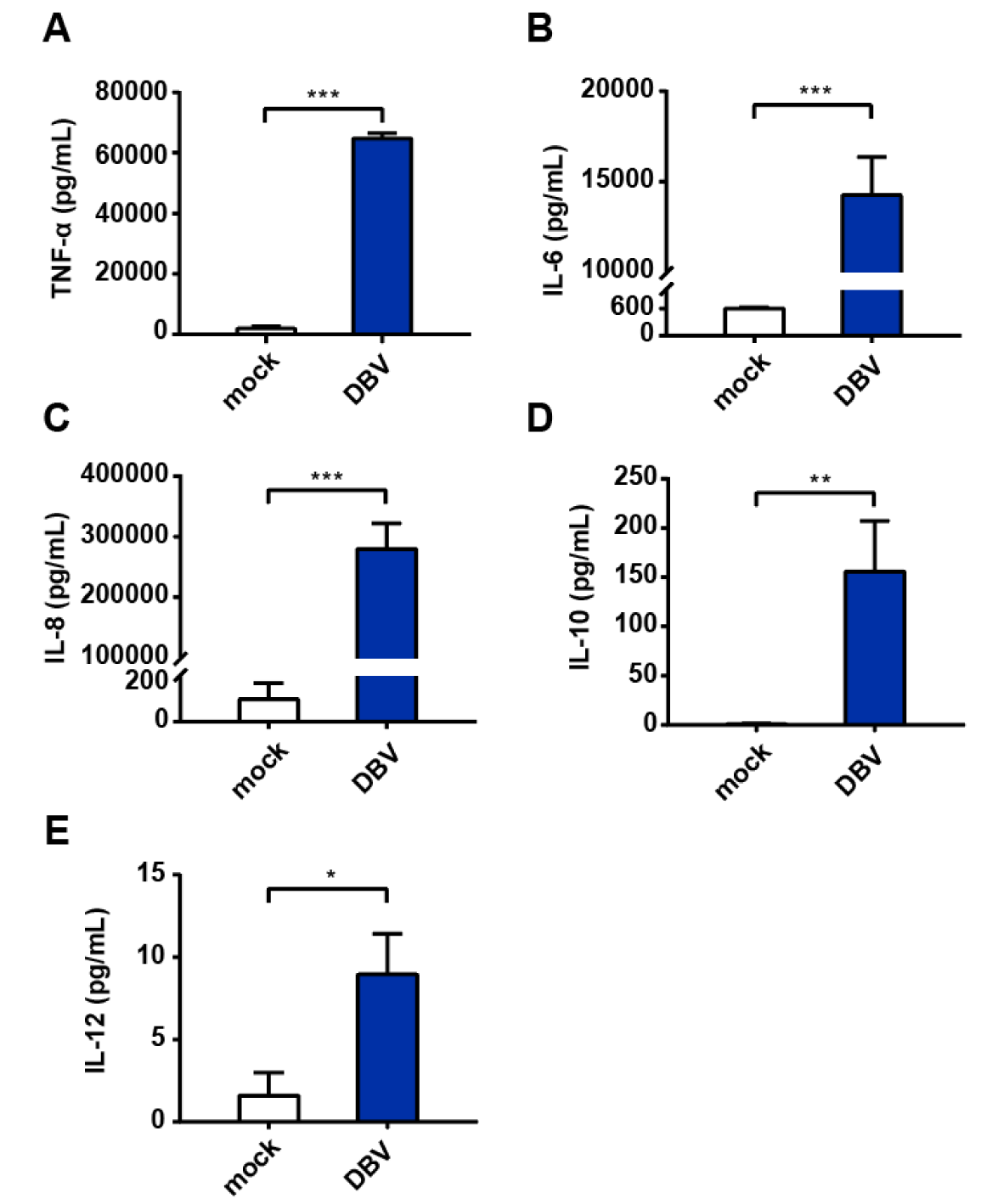

Supplement: S3 Fig — Macrophages were infected by DBV (MOI = 1) or were mock infected for 72 h. Then the supernatants were collected to measure (A) TNF-α, (B) IL-6, (C) IL-8, (D) IL-10 and (E) IL-12 production by ELISA. Data were mean values ± SD derived from the samples collected in triplicate. *P<0.05, **P<0.01, ***P<0.001, by two-tailed student t test. (TIF) [file pntd.0011488.s003.tif]

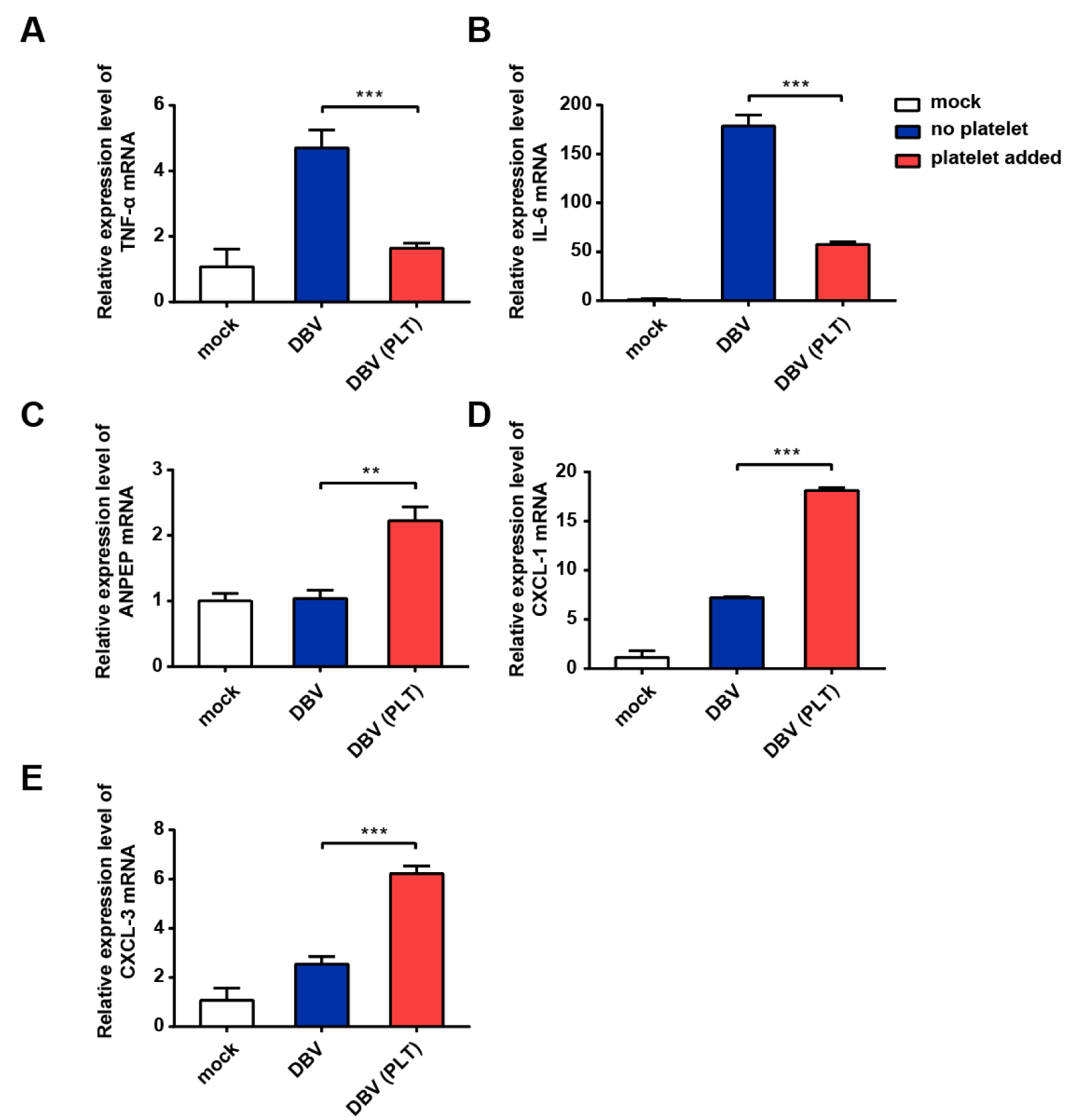

Supplement: S4 Fig — DBV-infected (MOI = 1) macrophages were co-incubated with or without platelets for 72 h. The mRNA expression levels of (A) TNF-α, (B) IL-6, (C) ANPEP, (D) CXCL-1 and (E) CXCL-3 were detected by RT-qPCR and analyzed by 2ΔΔCt method. Data were mean values ± SD derived from the samples collected in triplicate. *P<0.05, **P<0.01, ***P<0.001, NS = no statistical difference, by two-tailed student t test. (TIF) [file pntd.0011488.s004.tif]

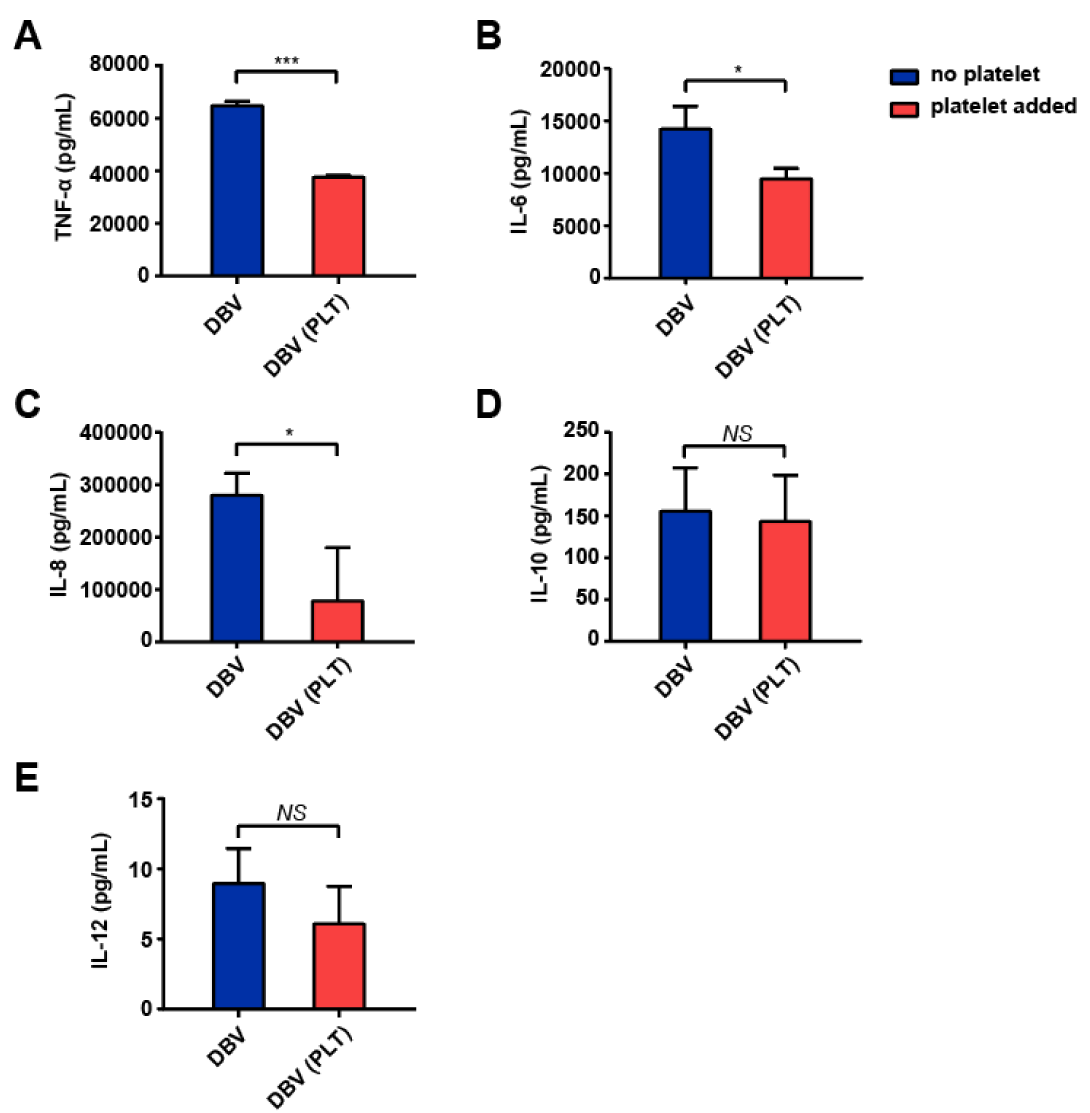

Supplement: S5 Fig — DBV-infected (MOI = 1) macrophages were co-incubated with or without platelets for 72 h. The cytokines levels of (A) TNF-α, (B) IL-6, (C) IL-8, (D) IL-10 and (E) IL-12 in the supernatants were analyzed by ELISA tests. Data were mean values ± SD derived from the samples collected in triplicate. *P<0.05, **P<0.01, ***P<0.001, NS = no statistical difference, by two-tailed student t test. (TIF) [file pntd.0011488.s005.tif]
